# A Review on Face Mask Recognition

**DOI:** 10.3390/s25020387

**Published:** 2025-01-10

**Authors:** Jiaonan Zhang, Dong An, Yiwen Zhang, Xiaoyan Wang, Xinyue Wang, Qiang Wang, Zhongqi Pan, Yang Yue

**Affiliations:** 1 School of Information and Communications Engineering, Xi’an Jiaotong University, Xi’an 710049, China; 2 Institute of Modern Optics, Nankai University, Tianjin 300350, China; 3 Drilling & Production Technology Research Institute, Chuanqing Drilling Engineering Company Limited, Guanghan 618300, China; 4 Angle AI (Tianjin) Technology Company Ltd., Tianjin 300450, China; 5 Department of Electrical & Computer Engineering, University of Louisiana at Lafayette, Lafayette, LA 70504, USA

**Keywords:** face mask detection, object detection, COVID-19

## Abstract

This review offers a comprehensive and in-depth analysis of face mask detection and recognition technologies, emphasizing their critical role in both public health and technological advancements. Existing detection methods are systematically categorized into three primary classes: feaRture-extraction-and-classification-based approaches, object-detection-models-based methods and multi-sensor-fusion-based methods. Through a detailed comparison, their respective workflows, strengths, limitations, and applicability across different contexts are examined. The review underscores the paramount importance of accurate face mask detection, especially in response to global public health challenges such as pandemics. A central focus is placed on the role of datasets in driving algorithmic performance, addressing key factors, including dataset diversity, scale, annotation granularity, and modality. The integration of depth and infrared data is explored as a promising avenue for improving robustness in real-world conditions, highlighting the advantages of multimodal datasets in enhancing detection capabilities. Furthermore, the review discusses the synergistic use of real-world and synthetic datasets in overcoming challenges such as dataset bias, scalability, and resource scarcity. Emerging solutions, such as lightweight model optimization, domain adaptation, and privacy-preserving techniques, are also examined as means to improve both algorithmic efficiency and dataset quality. By synthesizing the current state of the field, identifying prevailing challenges, and outlining potential future research directions, this paper aims to contribute to the development of more effective, scalable, and robust face mask detection systems for diverse real-world applications.

## 1. Introduction

In early 2020, the World Health Organization (WHO) classified coronavirus disease (COVID-19) as a transmissible epidemic [1]. Since its outbreak, it has posed a severe threat to global individual safety, with the subsequent emergence of various variants and mutations exacerbating the situation. Extensive research indicates that COVID-19 primarily spreads through droplets and aerosols during social interactions. Consequently, promoting the correct use of protective face masks by individuals is regarded as a crucial strategy to mitigate viral transmission [2]. In this context, to ensure that face-mask-wearing strategies are effectively implemented and to meet public health protection requirements, face mask detection technology has emerged as a prominent research focus. This technology not only assists in detecting whether individuals are wearing face masks but also assesses the properness of their wearing manner, thereby facilitating the intelligent management of public spaces and effectively preventing the sustained spread of epidemics [3]. Furthermore, to further reduce pandemic risks, it is often necessary to combine social distancing monitoring with routine temperature screening. As a result, research focused on face mask recognition and detection has significantly increased in recent years [4,5,6,7,8,9], aiming to enhance preventive and control measures against epidemics [10].

**Figure 1:**
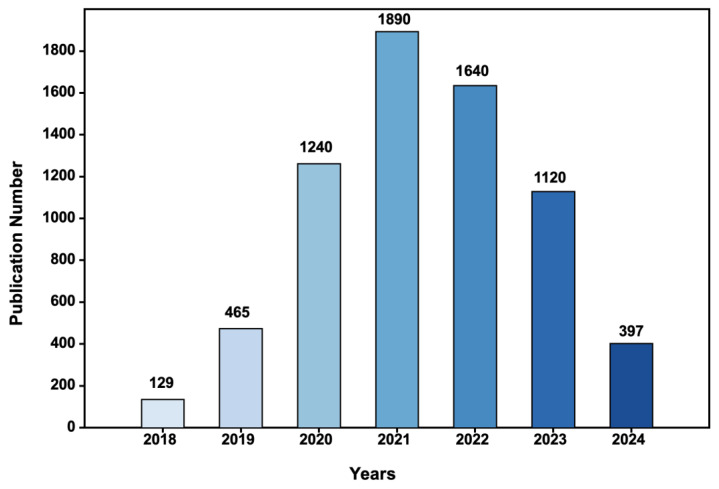
Publication number of face mask detection and recognition from 2018 to 2024 (source: Google Scholar).

Due to differing application scenarios and target requirements, face mask detection algorithms during the COVID-19 pandemic can be broadly categorized into two types. Firstly, there are those designed for environments such as checkpoints and entry/exit control points, where the distance between individuals and cameras is relatively close and the acquisition of high-quality images of single facial targets is enabled. Consequently, higher detection accuracy is required, typically employing traditional convolutional neural networks (CNNs) [11] for feature extraction and image classification [12,13,14,15,16,17,18,19,20,21,22,23,24]. Secondly, there are algorithms intended for public places with high foot traffic and dense crowds, where complex backgrounds and multiple interfering factors present greater challenges for detection tasks, especially with multi-scale facial targets, wherein small-scale faces are more prevalent. To address this, numerous researchers have proposed corresponding enhancement methods based on object detection models [25] to accommodate the detection needs of multi-scale face masks [15,16,17,18,19,20,21,22,23,24,25,26,27,28,29,30,31,32,33,34,35,36,37,38,39,40,41,42,43,44,45,46,47,48,49,50,51,52,53,54,55,56,57,58,59,60,61,62,63,64]. Figure 1 illustrates the number of relevant publications on face mask detection and recognition collected from 2018 to 2024. Concurrently, issues surrounding masked face recognition have also become increasingly prominent, with factors such as mask types, camera resolutions, and degrees of occlusion exacerbating the difficulty of detection and recognition. Moreover, aspects such as real-time video analysis and privacy protection have imposed higher demands on system design [65].

The rapid development of deep learning (DL) models and computer vision (CV) has provided robust technical support for achieving efficient and accurate face mask detection and facial recognition [66,67,68]. Not only are there face detection models such as multi-task cascaded convolutional neural networks (MTCNNs) [69], the OpenCV single-shot detector (OCVSSD), dual-shot face detectors (DSFDs) [70], RetinaFace [18] and BAIDU detectors [71], but also classical modular CNN-based models, including AlexNet [72], VGG [73], ResNet [74], SqueezeNet [75], DenseNet [76], GoogleNet [77], and MobileNet [78]. Additionally, numerous object detection algorithms have been developed, encompassing two-stage models like R-CNN [79], Fast R-CNN [80], and the Faster R-CNN [81] series, as well as one-stage models such as the You Only Look Once (YOLO) [82] series, the Shot MultiBox detector (SSD) [83], and RetinaNet [84], among other rapid frameworks. By leveraging these powerful model algorithms, face mask detection and recognition technologies have achieved significant maturity in practical applications.

Moreover, the utilization of enhanced artificial intelligence (AI) technologies not only enables (i) the real-time identification and tracking of target individuals across diverse scenarios, (ii) the monitoring of social distancing between individuals, and (iii) dynamic analysis and decision-making based on data from smartphones, cameras, and other sensing devices [85], but also facilitates the automated assessment of face mask usage. This effectively reduces the costs associated with manual inspections and the risks of cross-infection. In the context of escalating demands for public health safety and human health protection, face masks have transcended their role as mere protective equipment during pandemics. They now play a crucial role in industrial production, dust suppression, noise reduction, medical care, and other high-risk environments. In recent years, face masks have also been widely adopted in numerous non-pandemic settings. For instance, in environments with high levels of dust and harmful gases, such as the chemical, mining, and manufacturing industries, automated face mask detection can assist regulatory personnel in swiftly identifying and ensuring the proper usage of face masks by workers, thereby reducing the risks of occupational diseases and safety incidents [86]. Additionally, in medical and laboratory settings, the wearing of face masks can effectively minimize the transmission of pathogenic microorganisms, providing dual protection for both patients and healthcare workers [87]. It is foreseeable that, with the continuous emergence of innovative algorithms and hardware devices, face mask detection and recognition technologies will play an increasingly pivotal role in public health safety management and the control of other infectious diseases.

This paper provides an extensive and systematic analysis of face mask detection algorithms, highlighting significant advancements and research efforts in the field. The algorithms are categorized according to their model structures, with a thorough examination of the corresponding scenarios, advantages, and limitations for each approach. Furthermore, the paper addresses the ongoing challenges in the domain and proposes potential avenues for future research. The primary contributions of this review, distinguishing it from existing studies, are outlined as follows:

(1) A comprehensive evaluation of public datasets: This review offers an exhaustive categorization and evaluation of publicly available datasets for face mask detection, with a particular focus on their scale, diversity, and annotation granularity. By identifying critical challenges, such as data insufficiency and inherent biases, we provide actionable strategies to enhance dataset diversity, reduce bias, and improve fairness in the training and evaluation of face mask detection models. This contribution is novel in its comprehensive approach to dataset assessment, a subject which has been insufficiently explored in previous literature.
(2) The categorization and in-depth analysis of detection methods: This review classifies existing face mask detection methods into three primary categories: feature-extraction-and-classification-based approaches, object-detection-models-based methods and multi-sensor-fusion-based methods. Through a detailed analysis of their workflows, strengths, limitations, and appropriate application scenarios, we offer a clear, comparative technical overview that highlights the unique advantages and challenges of each approach. This classification, along with its analysis, provides novel insights into the strengths and trade-offs inherent in the choice of method, offering a valuable resource for researchers and practitioners.
(3) An exploration of multimodal techniques for enhanced detection: This review also investigates the use of multimodal techniques, such as depth and infrared imaging, in face mask detection. We explore their potential in addressing complex real-world environments, emphasizing their advantages in improving detection robustness under challenging conditions. Additionally, we identify and discuss the challenges associated with these techniques, including hardware cost, data fusion complexity, and privacy concerns. This contribution is significant as it bridges the gap between traditional visual-based methods and advanced multimodal approaches, offering novel perspectives for future face mask detection research.

The remaining sections of this review are structured as follows: Section 2 introduces and compares key datasets for face mask detection and recognition, focusing on their scale, annotation granularity, and applicability. Section 3 reviews detection methodologies, categorizing them into feature-extraction-and-classification-based approaches, object-detection-models-based methods and multi-sensor-fusion-based methods, while discussing emerging trends such as multimodal fusion and lightweight optimization. Section 4 provides a detailed discussion of datasets and methods, highlighting key challenges, trade-offs, and future research directions. Finally, Section 5 summarizes the review, emphasizing dataset design, methodological evolution, and multimodal integration, while proposing pathways to enhance robustness and adaptability in real-world applications.

## 2. Datasets

**Figure 2:**
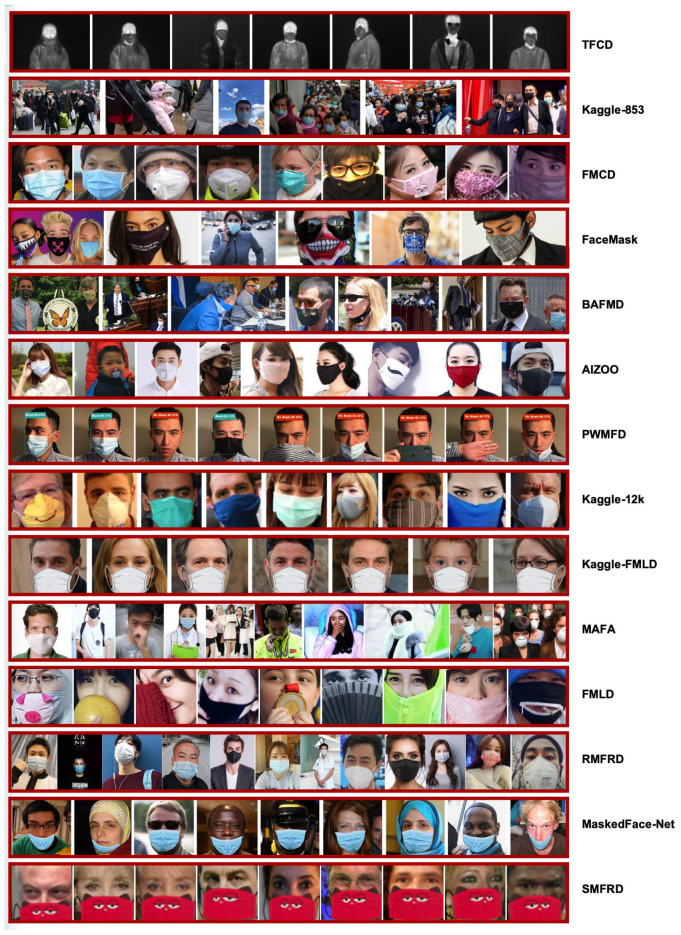
Example images of the different datasets used in the COVID-19 era.

Over the past few years, many face mask datasets have been introduced to solve several tasks related to COVID-19. During the pandemic, the demand for suitable large-scale face mask image collections increased significantly. Consequently, new datasets targeting face mask detection and recognition, masked face detection and recognition, and other related issues were introduced. In Table 1, we summarize the primary datasets associated with COVID-19, compare their characteristics, and present some example images in Figure 2.

MAFA [88] is the first large-scale face mask dataset to have been released, comprising 35,806 face images with face masks. This dataset includes six annotation attributes: face, eyes, and face mask bounding coordinates; head pose; face mask coverage; and four different types of face masks. Notably, some face masks in the data are worn incorrectly, such as by not covering the nose. Consequently, face mask detection models developed using this dataset are generally considered less suitable for surveillance applications aimed at preventing the transmission of COVID-19.

However, the original annotations of MAFA are not suitable for training effective detectors to distinguish between correctly and incorrectly worn face masks. Therefore, FMLD [8] re-annotated the MAFA images. FMLD comprises three categories of images: 29,532 images of correctly worn face masks, 1528 images of incorrectly worn face masks, and 32,012 images of faces without face masks. In addition to face mask annotations, FMLD also includes bounding coordinates of faces, as well as labels for gender, ethnicity, and the pose of each face.

FMCD [89] is derived from the MAFA dataset by cropping and filtering face images with face masks and standardizing their sizes to 224 × 224 pixels. The dataset categorizes face masks into two classes: qualified masks (OK masks) and unqualified masks (NG masks). Qualified face masks include N95 masks and disposable medical masks, comprising a total of 1361 images. Unqualified face masks include sponge masks, cloth masks, and scarves, comprising a total of 1880 images. The entire face mask classification dataset contains 3241 images, which are utilized for training and evaluating face mask classification algorithms.

WearMask [90] developed a serverless edge face detection tool using the Wider Face and MAFA datasets. Their dataset includes 4065 images from MAFA, 3894 images from Wider Face, and an additional 1138 images sourced from the internet, amounting to a total of 17,532 face images with corresponding bounding boxes.

In the PWMFD [91] dataset, the collectors considered three categories of labels: correctly worn face masks, incorrectly worn face masks, and no face mask. The dataset comprises a total of 7695 correctly worn face masks, 10,471 face images without face masks, and 366 incorrectly worn face masks. These include 3615 newly collected images, 2581 re-annotated images from MAFA, 2951 images from Wider Face, and 58 face images from RMFRD.

FaceMask [92] dataset was constructed by collecting 4866 images from Google using keywords like “people wearing face mask” and “crowds during coronavirus”. The images depict people of various ages in indoor and outdoor settings, with individual faces, partially occluded faces, and crowded scenes. Duplicate images were removed using AntiDupl.NET, and annotations were made using LabelImg, categorizing images into “Mask” and “No_Mask” classes. This dataset provides a valuable resource for developing and testing face mask detection algorithms.

The BAFMD [93] dataset, which contains 6264 images from Twitter and over 16,000 face bounding boxes with and without face masks, was collected with the aim of creating a dataset that minimizes potential bias in terms of ethnicity, age and gender.

The MaskedFace-Net [94] dataset consists of the Correctly Masked Face Dataset (CMFD) and the Incorrectly Masked Face Dataset (IMFD) subsets. The MaskedFace-Net was constructed using the Masked-Face Deformable model and contains a total of 137,016 images.

Ref. [95] constructed 500,000 simulated masked face images from 10,000 subjects using an artificial mask generation tool. A total of three new datasets, named Masked Face Detection Dataset (MFDD), Real World Masked Face Recognition Dataset (RMFRD), and Simulated Masked Face Recognition Dataset (SMFRD), are included to study the performance of masked face detection and face recognition under mask occlusion.

COVID-19 TFCD [96] collected a small thermal mask dataset containing 250 images belonging to 20 participants using thermography for COVID-19 related applications.

In addition to this, there are a number of other online dataset repositories. For instance, in Kaggle-853 there are 853 images, containing 4080 faces in 3 face categories (present/absent/improperly worn). Kaggle-12k publishes about 12 k images of faces belonging to two categories: masked and unmasked. This dataset varies in terms of resolution, mask type and different populations. Another dataset on Kaggle, called the Face Mask Lite Dataset (Kaggle-FMLD), contains 10,000 face images of human workers. The AIZOO dataset is a publicly available mask face detection dataset and annotation work published by private companies.

In this review, based on the provided dataset characteristics, we provide a more profound academic discussion of the current COVID-19-related masked face datasets in terms of the dimensions of data size, data type, annotation accuracy and diversity, application scenario applicability, and fairness. The combing of these dimensions helps researchers to select and combine appropriate datasets in a more targeted way during model development and task implementation, thus effectively enhancing the generalization ability and practical value of the model.

(1) The richness and diversity of dataset sizes: Today’s existing datasets show great diversity in scale, ranging from as small as only a few hundred images (e.g., about 250 images for TFCD) to as large as tens or even hundreds of thousands of images (e.g., MAFA, FMLD, RMFRD, MaskedFace-Net, SMFRD). This multivariate distribution from small to large scale not only facilitates rapid prototyping and exploration under low-resource conditions, but also lays the data foundation for high-complexity training and generalization performance testing of deep models. Researchers can flexibly choose and combine datasets of different sizes according to their own research stages and task attributes, in order to strike a balance between computational overhead and model performance.
(2) Complementary advantages of real images and synthetic datasets: The data sources are both real-world captured images (e.g., MAFA, FMLD, RMFRD) and synthetic and generated images (e.g., BAFMD, Kaggle-FMLD, MaskedFace-Net, SMFRD). Real datasets better reflect the variability and complexity of the actual environment and improve the robustness of the model in real-world scenarios, while synthetic datasets ensure the consistency and diversity of annotations through a controlled data generation process, providing a stable foundation for model pre-training, data enhancement, and domain self-adaptation. Combining the two organically helps to further enhance the applicability and performance ceiling of the model.
(3) The increasing granularity of labeling versus task complexity: The dataset annotation extends from the initial binary categorization (masked/unmasked) to more complex category and attribute annotations, such as considering wrongly worn (wrongly worn), diverse mask types, and facial keypoint localization (e.g., FMLD, WearMask, PWMFD). Fine-grained annotations help researchers to deeply explore mask-wearing behavior and its impact on face recognition and detection performance, and provide support for subsequent attribute prediction, bias analysis, segmented scene response, and more fine-grained tasks (e.g., distinguishing between different types of mask materials and wearing styles).
(4) Real-world scenario applicability with domain-specific applications: Most datasets introduce diverse scenes, lighting conditions, crowd composition and ingestion angles (e.g., FaceMask, AIZOO, WearMask, etc.) into the data collection and screening, so as to make the data more suitable for the actual application environment. This is especially critical for face monitoring during epidemics, security monitoring in public places, and personnel protection detection in healthcare scenarios. Researchers can select datasets based on the specific needs of their application domains to ensure that the constructed models will perform robustly in field deployments.
(5) Data diversity and equity concerns: Some datasets (e.g., BAFMD) emphasize a balanced distribution of different races, genders, and ages in their data collection and labeling, reflecting the growing attention of academics to the issue of potential bias and fairness in datasets. Ensuring that datasets are sufficiently diverse and balanced can help reduce model performance bias in specific populations or particular scenarios, thereby enabling more inclusive and equitable decision-making in real-world applications.

In synthesis, these COVID-19-related mask face datasets provide a multi-layered and complementary resource base for the development of current research and applications. By understanding and utilizing the differences and advantages of the datasets in terms of size, data type, annotation granularity, diversity of real-world scenarios, and fairness, researchers and engineering practitioners can make significant advances in constructing smarter, more robust, and fairer mask-wearing detection and recognition systems.

## 3. Methods for Face Mask Detection and Recognition

The development of mask detection and recognition technologies has been largely propelled by continuous innovations in CV and DL. Broadly speaking, existing methodologies can be divided into two primary technical paradigms: one grounded in feature extraction and classification, typically relying on either traditional or deep-learning-based techniques to extract discriminative features from images before passing them to a classifier to determine mask-wearing status, and another that adopts a generalized object detection framework, treating masks as targets whose locations and categories are identified in a single step, thereby enhancing detection efficiency and adaptability [12,13,14,15,16,17,18,19,20,21,22,23,24]. While the former approach tends to be more straightforward in terms of implementation and interpretability—particularly when precise mask-wearing analysis or additional facial feature inspection is required—recent advances in object detection have enabled the latter approach to excel in crowded or complex backgrounds, thanks to notable progress in multi-object and multi-scale detection [15,16,17,18,19,20,21,22,23,24,25,26,27,28,29,30,31,32,33,34,35,36,37,38,39,40,41,42,43,44,45,46,47,48,49,50,51,52,53,54,55,56,57,58,59,60,61,62,63,64]. This section provides a comprehensive overview of these two paradigms, illustrates representative models, and discusses relevant lightweight optimization strategies and emerging techniques, offering a systematic reference for the application of mask detection and recognition across diverse scenarios and tasks.

### 3.1. Feature-Extraction-and-Classification-Based Methods

#### 3.1.1. Traditional Feature Extraction

In early face mask detection research, due to the limited widespread application and insufficient computational power of DL, researchers often relied on traditional feature engineering and machine learning pipelines to classify the status of face mask usage. Specifically, the general workflow of such methods typically includes the following steps: first, performing feature extraction or local scanning on the entire image to capture key features related to the face mask region; then, inputting these extracted features into machine learning classifiers (such as support vector machines (SVMs), random forests (RFs), or logistic regression (LR)) for training and inference, thereby determining whether there are individuals wearing face masks in the image and assessing the correctness of their mask usage. Refs. [13,14,23] employed traditional machine learning methods, including the use of SVM, to identify the optimal boundary hyperplane for distinguishing between different classes, as well as decision trees to recursively select the most informative attributes for data segmentation and construct hierarchical tree structures. These approaches are utilized to detect whether individuals are wearing face masks during the COVID-19 pandemic.

However, these methods also exhibit significant limitations. On one hand, traditional features are often sensitive to variations in lighting, pose changes, and partial occlusions, which can lead to reduced stability and robustness in feature extraction. On the other hand, these methods largely lack dedicated detection of face regions or the capability for precise localization of face masks. Consequently, when faces in images appear with significant angle rotations or severe occlusions, classification accuracy markedly decreases. Furthermore, in scenarios with dense crowds or substantial variations in face scales, relying solely on entire image or local scanning approaches may fail to adequately capture the critical details necessary to distinguish whether a face mask is worn, resulting in increased rates of false negatives or false positives.

Precisely because of these challenges, as CNNs have demonstrated powerful feature learning capabilities in image recognition tasks, traditional feature extraction methods have gradually been supplanted or integrated with subsequent DL models.

Overall, face mask detection methods based on traditional feature extraction and classical classifiers are simple to implement and easy to understand. However, their accuracy and robustness are often inferior compared with methods based on face detection models or DL approaches. Therefore, in practical applications, researchers commonly combine such traditional methods with more advanced detection modules or lightweight deep networks to balance algorithm interpretability and detection performance.

#### 3.1.2. Facial Feature Extraction

In the context of face mask detection, integrating “face detection” with “face mask recognition” is a relatively intuitive and effective approach. The core methodology involves initially and accurately locating the face region of interest (ROI) within the entire image, followed by inputting this ROI into a classification model to determine both the presence of a face mask and the correctness of its usage. Compared with the process of performing face mask detection directly on the entire image, this two-stage pipeline leverages the maturity and accuracy of face detection technologies more effectively, enabling high-resolution feature extraction focused on the localized face region during the classification phase.

**Figure 3:**
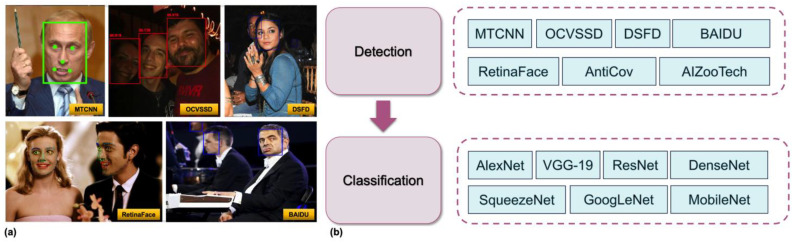
(**a**) Some widely used face detection models. (**b**) Two-step workflow diagram for using a face detector and classifier for face mask detection and recognition.

During the first stage of face detection, researchers widely employ specialized face detection models, such as MTCNN [72] or RetinaFace [18], as shown in Figure 3a. These models utilize multi-level or end-to-end convolutional network architectures to simultaneously achieve face bounding box localization and keypoint detection (e.g., eyes, nose, mouth corners), thereby providing robustness against non-frontal angles and varying lighting conditions. For instance, MTCNN employs a cascade of three sub-networks (P-Net, R-Net, O-Net) to filter candidate boxes at different scales, progressively refining face localization and correcting region coordinates. In contrast, RetinaFace adopts a single-stage detection approach prevalent in the object detection domain, integrating feature pyramid networks (FPNs) with self-supervised multi-task learning to achieve high-precision detection of multi-scale faces. Regardless of the detection model utilized, the final output invariably consists of one or more bounding boxes that delineate the spatial positions of faces within the image.

**Figure 4:**
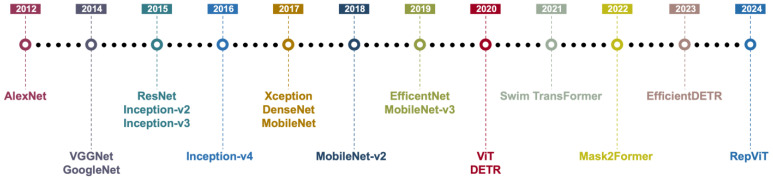
Evolution of deep neural network modules.

In the second stage of face classification (or face mask classification), the predominant practice involves inputting the cropped face ROIs into pre-trained deep CNNs for feature extraction and classification. Examples of such CNN architectures include AlexNet [75], VGG [76], ResNet [77], SqueezeNet [78], DenseNet [79], GoogleNet [80], and MobileNet [81]. Figure 4 shows the evolution of deep neural network modules. Depending on the network size and downstream application requirements, researchers may opt for lightweight models with fewer parameters suitable for embedded or mobile environments (e.g., MobileNet) to achieve higher accuracy. During this process, some studies further analyze the degree of occlusion of facial keypoints (e.g., whether the nose is exposed, whether the mask covers both the nose and mouth) to make more nuanced judgments regarding the correctness of mask wearing. To mitigate detection challenges posed by multiple poses, varying lighting conditions, or partial occlusions, certain systems perform face alignment or keypoint correction prior to inputting the ROI, thereby enhancing the classification model’s tolerance to occlusion and deformation.

Based on the aforementioned workflow, numerous combination schemes have been proposed. For example, [19] introduces a two-stage CNN architecture designed to detect faces with correctly worn and non-worn face masks. In the first stage, a pre-trained RetinaFace model is employed for face detection. The second stage utilizes a lightweight CNN classifier based on MobileNet to classify the detected faces regarding their face mask-wearing status. Furthermore, a centroid tracking algorithm is integrated within the video stream to enhance the stability of detections. Ref. [8] selected multiple pre-trained face detection models, including MTCNN, OCVSSD, DSFD, RetinaFace, the Baidu detector, AntiCov, the AIZooTech detector, as well as various CNN models for classification tasks, and evaluated them on a test set, as shown in Figure 3b. Among these, the RetinaFace model demonstrated the most stable performance in detecting faces with face masks, achieving an AP50 score of 86.61% and an overall AP of 92.93% on the entire dataset. All tested classification models (including AlexNet, VGG-19, ResNet series, SqueezeNet, DenseNet, GoogleNet, and MobileNet) attained over 97% accuracy in the task of correctly identifying face mask placement. Additionally, [8] developed a comprehensive recognition pipeline by integrating the best detection model (RetinaFace) and classification model (ResNet-152). This model exhibited outstanding performance in the task of detecting correct face mask placement, with an AP50 score of 90.75% and an AP40 score of 95.72%, significantly outperforming existing face mask detection models. [8] delineated the comprehensive workflow of a face mask detection model, which initially performs face detection followed by the classification of face mask usage.

These two-stage approaches, which combine face detection and face mask classification, offer several advantages: on one hand, leveraging the precision of face detection models in localization and alignment yields cleaner and more focused face regions; on the other hand, compared with general object detection models, this methodology often provides greater scalability in assessing the correctness of mask wearing and capturing mask-related features in facial positions. However, it is important to note that, unlike “one-step” object detection methods, two-stage approaches typically require multiple image convolution operations, which may present efficiency challenges when processing dense crowds or high-resolution videos. Consequently, subsequent research continues to explore optimization techniques such as model pruning, quantization, and knowledge distillation for both detectors and classifiers to balance detection speed and accuracy. By selecting appropriate face detection models and classification networks tailored to specific application scenarios, researchers can construct more flexible and competitive face mask detection systems.

### 3.2. Object-Detection-Model-Based Methods

**Figure 5:**
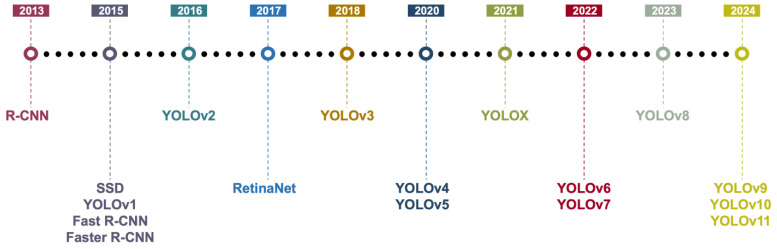
Development of DL-based object detection models.

In addition to feature-extraction-and-classification-based methods, face mask detection approaches based on object detection models have also achieved significant progress in recent years [15,16,17,18,19,20,21,22,23,24,25,26,27,28,29,30,31,32,33,34,35,36,37,38,39,40,41,42,43,44,45,46,47,48,49,50,51,52,53,54,55,56,57,58,59,60,61,62,63,64]. Object detection models, by simultaneously performing localization and classification of targets within an image, can efficiently handle multi-object and multi-scale detection tasks, making them particularly suitable for scenarios with dense crowds and complex backgrounds. Figure 5 shows the development of DL-based object detection models, which encompass a range of advanced object detection models, including the R-CNN series, the YOLO family of models, and the SSD framework, which collectively represent significant milestones in the evolution of deep learning-based object detection methodologies. With the continuous evolution of DL technologies, single-stage and two-stage detectors have each demonstrated unique advantages: single-stage detectors, characterized by their high inference speed, are well-suited for real-time monitoring and for applications with limited resources, whereas two-stage detectors excel in scenarios requiring precise identification and high accuracy due to their superior detection precision. Moreover, to meet the real-time and computational resource constraints of practical applications, researchers have been exploring lightweight and improved methodologies. Techniques such as model pruning, quantization, and knowledge distillation are employed to optimize the performance and efficiency of detectors. The following subsections will provide a detailed introduction to the specific applications and development trends of single-stage object detectors, two-stage object detectors in the context of face mask detection.

#### 3.2.1. Based on Single-Stage Object Detection

In the context of face mask detection, single-stage object detection methods have garnered widespread attention due to their simplicity and efficiency. Compared with two-stage detection methods, single-stage detectors integrate both bounding box regression and class identification within the same network, thereby eliminating the repetitive processes of generating candidate proposals and performing fine-grained classification. This integration significantly enhances detection speed.

Typical single-stage detectors include the SSD [86], which adapts to multi-scale face scenarios by predicting bounding boxes at multiple scales and performing multi-class classification outputs. The SSD training objective is extended to handle multiple object categories. Let $x_{ij}^{p}=\left\{1,0\right\}$ be an indicator for matching the i-th default box to the j-th ground truth box of category p. In the matching strategy above, we can have $\sum_{i}x_{ij}^{p}\;\geq \;1$. The overall objective loss function is a weighted sum of the localization loss (*loc*) and the confidence loss (*conf*), which is shown in the following Equation (1):

(1) $$L\left(x,c,l,g\right)=\frac{1}{N}\left(L_{conf}\left(x,c\right)+\alpha L_{loc}\left(x,l,g\right)\right)$$

where *N* is the number of matched default boxes, and the localization loss is the smooth *L*1 loss between the predicted box (*l*) and the ground truth box (*g*) parameters. Similar to Faster R-CNN, they regress to offset for the center of the bounding box and for its width and height. The confidence loss is the softmax loss over multiple class confidences (*c*) and the weight term α is set to 1 by cross validation.

Ref. [59] proposed an intelligent internet of things (IoT)-based face mask detection model that utilizes DL methodologies. Specifically, the model integrates the SSD with the hybrid DL classifier MobileNet. Additionally, parameter optimization is performed using the Adaptive Swarm Marine Fuzzy Optimization (ASMFO) algorithm to enhance the efficiency and accuracy of face mask detection. Ref. [52] employed an enhanced SSD integrated with the VGG-16 architecture to detect face mask usage. Compared with CNNs, the SSD achieves an accuracy of 92.25%, significantly surpassing the 82.6% accuracy obtained by the CNN.

**Figure 6:**
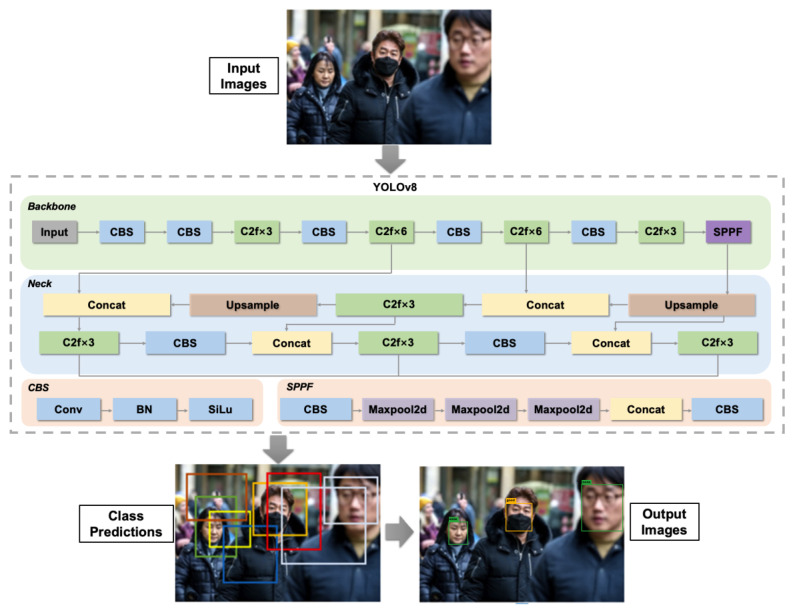
Single-stage face mask detection model based on YOLOv8 architecture.

The YOLO series (v3, v4, v5, v6, v7, v8, etc.), which partition the image into grids and directly regress bounding boxes and confidence scores, offer high inference efficiency and active model updates, with lightweight versions (e.g., YOLOv6-tiny) being more suitable for mobile or real-time monitoring applications. Ref. [46] leveraged the YOLOv8 algorithm, incorporating a transformer-based architecture and advanced training techniques such as knowledge distillation and pseudo-labeling, to enhance the accuracy and efficiency of object detection. By integrating the facial mask dataset (FMD) and the medical mask dataset (MMD), experiments were conducted to validate the performance of YOLOv8 in detecting and classifying masked faces. Notably, the YOLOv8m model achieved an average precision (mAP) of 99.1% for the “good” category (correctly worn face masks) and an overall average precision of 78.4%, surpassing prior studies and models. Convolution, batch normalization, and activation functions for the YOLOv8 architecture are the three basic components that make up the CNNs that are depicted in Figure 6, where the yellow box represents the mask being worn correctly and the green box represents the mask not being worn.

The core structure of YOLOv8 is quite like that of YOLOv5, except for the C3 module, which has been replaced with the C2f module. This module is derived from the CSP idea. YOLOv8’s C2f module was produced by using the ELAN concept from YOLOv7 and combining it with C3. This was carried out in order to develop the module. This integration was undertaken to improve YOLOv8’s gradient flow information without jeopardizing its lightweight design in any way. The dominant SPPF module was used throughout the entirety of the final stage of the backbone architecture. After this, a sequential application of three waxpools, each of which had a size of 5 by 5 inches, was carried out. After that, the output of each layer was concatenated to ensure the accurate detection of objects at various scales while keeping a lightweight design. This was accomplished without sacrificing accuracy. Below is the YOLO loss function in Equation (2):

(2) $$YOLO\;Loss\;Function\;\;{=\lambda }_{coord}\sum_{i=0}^{s^{2}}\sum_{j=0}^{B}{∥}_{ij}^{obj}\left[{\left(x_{i}-{\hat{x}}_{i}\right)}^{2}+{\left(y_{i}-\hat{y_{i}}\right)}^{2}\right]\;+{\;\lambda }_{coord}\sum_{i-0}^{s^{2}}\sum_{j=0}^{B}{∥}_{ij}^{obj}\left[{\left(\sqrt{\omega_{i}}-\sqrt{{\hat{\omega }}_{i}}\right)}^{2}+{\left(\sqrt{h_{i}}-\sqrt{{\hat{h}}_{i}}\right)}^{2}\right]\;+\sum_{i-0}^{s^{2}}\sum_{j=0}^{B}{∥}_{ij}^{obj}{\left(C_{i}-{\hat{C}}_{i}\right)}^{2}+\lambda_{noobj}\sum_{i-0}^{s^{2}}\sum_{j=0}^{B}{∥}_{ij}^{noobj}{\left(C_{i}-{\hat{C}}_{i}\right)}^{2}\;+\sum_{i-0}^{s^{2}}{∥}_{ij}^{noobj}\sum_{c\epsilon classes}{\left(p_{i}\left(c\right)-{\hat{p}}_{i}\left(c\right)\right)}^{2}$$

where ${∥}_{ij}^{obj}$ indicates whether the object appears in cell i and ${∥}_{ij}^{obj}$ denotes that the jth bounding box predictor in cell i is responsible for the prediction. Next, $\left(\hat{x},\hat{y},\;\hat{\omega },\;\hat{h},\hat{c},\;\hat{p}\right)$ is implemented to express the anticipated bounding box’s center coordinates, width, height, confidence, and category probability. This experiment employed the ${\;\lambda }_{coord}$ to 0.5, demonstrating that the width and height errors are less useful in the computation. To mitigate the effect of numerous vacant grids on the loss value, ${\;\lambda }_{noobj}$ = 0.5 is utilized.

The findings not only demonstrate the high efficiency of the YOLOv8m model in detecting medical face masks, but also provide new directions for future research, including exploring the application of DL models for recognizing partially obscured faces in static images and videos, as well as employing explainable artificial intelligence (XAI) for medical face mask recognition.

In practical applications, to better adapt single-stage detection methods to face mask detection scenarios, researchers often introduce the following improvements: firstly, by integrating FPN or customized feature fusion modules to enhance multi-scale detection, which significantly improves the detection of small face targets in distant or low-resolution scenes; secondly, by employing data augmentation techniques such as random cropping, scaling, translation, and rotation, as well as hard negative mining strategies, the adaptability of models can be effectively enhanced, and sensitivity to complex backgrounds and occlusions can be reduced; furthermore, through methods such as pruning, quantization, knowledge distillation, and the incorporation of ghost modules to streamline mainstream network architectures, it is possible to reduce inference latency while maintaining detection accuracy, making these models suitable for high-frame-rate monitoring or devices with limited computational resources; lastly, during the post-processing stage, adopting more sophisticated non-maximum suppression (NMS) or soft-NMS strategies can minimize the occurrence of duplicate detections for overlapping bounding boxes, thereby further enhancing the reliability of the final detection outcomes.

Overall, single-stage detectors, leveraging a “single regression and classification” framework, demonstrate notable speed advantages in face mask detection, particularly exhibiting significant potential in applications with high foot traffic and stringent real-time monitoring requirements. When combined with multi-scale feature fusion and lightweight optimizations, single-stage models not only enhance the ability to capture small-scale face mask targets but also facilitate deployment on devices with limited computational resources. Consequently, they have become one of the prominent directions in contemporary face mask detection research and applications.

#### 3.2.2. Based on Two-Stage Object Detection

Two-stage object detection models typically accomplish detection tasks through two primary steps: “Region Proposal Generation” and “Refined Classification and Regression”. Compared with single-stage detection methods, this coarse-to-fine detection process, although relatively slower in inference speed, achieves higher accuracy in target localization and class identification. Consequently, two-stage models exhibit exceptional performance in scenarios that demand high detection precision or require the fine-grained recognition of small-scale targets. Specifically, for face mask detection, two-stage approaches can effectively reduce both false negatives and false positives in crowded or high-resolution environments, making them particularly suitable for application domains with stringent accuracy requirements, such as medical facilities, large public spaces, and security surveillance.

When encountering difficult scenes, such as those at multiple scales, those with small targets and those that are crowded, the R-CNN series has a strong advantage in detection accuracy. In 2014, Girshick et al. [82] proposed a two-stage R-CNN model for the first time by improving on the basis of a convolutional neural network, which used AlexNet [75] for feature extraction, and the final MAP was also much improved over the traditional method. Girshick et al. [83] introduced a spatial pyramid pooling network on this basis and proposed the Fast R-CNN model. After the previous iteration, the Faster R-CNN [84] network was proposed in 2017. From a convolutional neural network to Faster R-CNN network, the process of target detection based on DL has become increasingly streamlined, accurate and fast.

**Figure 7:**
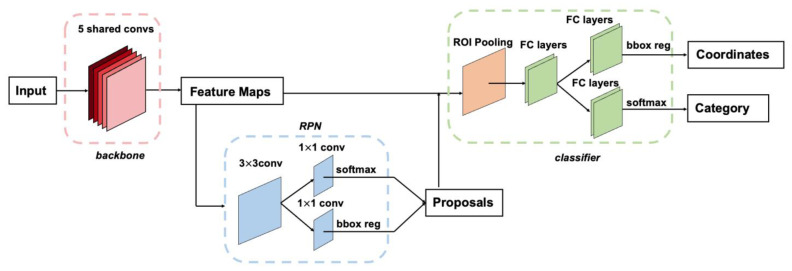
Algorithm flowchart of Faster R-CNN.

As illustrated in Figure 7, the Faster R-CNN algorithm comprises the following steps: first, the input image is passed through the backbone network to extract image features, producing feature maps that are shared for subsequent use in the region proposal network (RPN) and ROI pooling layers. The RPN is responsible for generating region proposals. This network employs a softmax function to determine whether anchors are classified as positive or negative and further refines the anchors through bounding box regression to obtain precise proposal boxes. The ROI pooling layer takes the feature maps and proposals as input, extracting proposal feature maps by integrating these inputs. These feature maps are then forwarded to the fully connected layers for classification of the target object. The classifier predicts the class of the proposals using the proposal feature maps processed through the fully connected layers. Concurrently, bounding box regression is performed once more to determine the final precise locations of the detection boxes. The loss function for an image is defined as in Equation (3):

(3) $$\;L\left(\left\{p_{i}\right\},\left\{t_{i}\right\}\right)=\frac{1}{N_{cls}}\sum_{i}L_{cls}\left(p_{i},p_{i}^{*}\right)+\lambda \frac{1}{N_{reg}}\sum_{i}p_{i}^{*}L_{reg}(t_{i},t_{i}^{*})$$

here, i is the index of an anchor in a mini-batch and $p_{i}$ is the predicted probability of anchor i being an object. The ground-truth label $p_{i}^{*}$ is 1 if the anchor is positive and is 0 if the anchor is negative. $T_{i}$ is a vector representing the four parameterized coordinates of the predicted bounding box, and $t_{i}^{*}$ is that of the ground-truth box associated with a positive anchor. The classification loss $L_{cls}$ is the log loss over two classes (object versus not object). For the regression loss, we use $L_{reg}\left(t_{i},t_{i}^{*}\right)=R\left(t_{i},t_{i}^{*}\right)$, where R is the robust loss function (smooth $L_{1}$ The term $p_{i}^{*}L_{reg}$ means the regression loss is activated only for positive anchors ($p_{i}^{*}=1$) and is disabled otherwise ($p_{i}^{*}=0$). The outputs of the cls and reg layers consist of $\left\{p_{i}\right\}$ and $\left\{t_{i}\right\}$, respectively.

In face mask detection applications, two-stage object detectors often better accommodate a variety of requirements. On one hand, by combining RPN with multi-stage feature extraction, these models can more precisely locate details such as the degree of facial occlusion and the edges of face masks, enabling fine-grained detection and localization. On the other hand, in common high-resolution surveillance videos, two-stage methods exhibit stronger capabilities in capturing distant or small-scale face targets, thereby effectively reducing the miss detection rate. Additionally, these methods can effortlessly extend to multi-task learning modules (such as keypoint detection or semantic segmentation) atop the traditional detection pipeline, thereby extracting more comprehensive information regarding mask-wearing methods and occluded regions.

### 3.3. Multi-Sensor-Fusion-Based Methods

In face mask detection and recognition tasks, single visible-light images often struggle to maintain high robustness and accuracy in complex scenarios due to limitations such as lighting variations, occlusions, and environmental interference. To address these challenges, researchers have increasingly integrated infrared images, depth images, or other sensor data with visible-light images, leveraging multimodal fusion to enhance overall system performance. For instance, in extreme environments, such as low-light or strong backlight conditions, infrared images can assist in capturing facial thermal radiation features, compensating for the poor performance of visible-light sensors in such scenarios. Similarly, depth images can provide three-dimensional geometric information, aiding in distinguishing real faces from flat disguise masks and improving the understanding of spatial relationships between the face mask and key facial regions. By incorporating volumetric or distance measurements, depth images can also assist in determining whether a face mask is properly worn and in detecting details such as partial slippage or improper placement.

In the practical implementation of multi-sensor fusion, some studies employ hardware setups such as binocular or multi-camera systems, combining visible-light and infrared cameras to capture data concurrently. Subsequently, at the algorithmic level, techniques such as point cloud registration, image alignment, and frame synchronization are utilized to preprocess and integrate multimodal data. These integrated data are then processed using multi-stream CNNs or transformer architectures to extract features from different modalities, which are subsequently combined at the feature fusion layer or the decision layer for comprehensive judgment. For the specific task of face mask detection, researchers typically focus on multidimensional factors, such as facial contours, deformations in the nose and mouth regions, and anomalies in thermal distribution, complementing these with texture details from visible-light images for enhanced analysis. This approach not only significantly improves detection accuracy in scenarios with poor lighting or partial occlusion but also provides a richer basis for identifying various specialized types of face masks, such as transparent masks, masks with breathing valves, or medical-grade protective masks.

TFCD [96] employed a long-wave infrared (LWIR) thermal imaging camera to capture images. This thermal imaging technology detects thermal radiation emitted by the human body, enabling facial detection under various environmental and lighting conditions. It maintains a high recognition rate even when the face is partially obscured by a face mask or viewed from extreme angles. The dataset comprises 250 images from 20 participants, illustrating diverse facial rotation angles and different facial coverings, such as face masks and glasses. This approach enabled the research team to develop a facial detection system operable under non-visible light conditions, which holds significant importance for contactless temperature monitoring and facial recognition during public health crises, such as the COVID-19 pandemic.

Compared with traditional optical cameras, depth cameras possess inherent advantages that make them highly reliable and resilient in low-light and dark environments. Unlike optical cameras, which may be influenced by ambient light, depth cameras maintain stability and robustness even in challenging lighting conditions. Furthermore, the 3D data obtained from depth camera photography offers a more precise representation of the real world. Leveraging fast imaging depth cameras enables accurate and rapid differentiation of face mask detection for individuals.

Ref. [97] presents a method that utilizes spatial and frequency features extracted from depth images captured by a time-of-flight (ToF) camera for face mask detection. The obtained results are classified into three categories: not wearing a mask, wearing a surgical mask, and wearing an N95 mask. By optimizing the spatial and frequency characteristics of the depth profile of the face, these three cases can be easily differentiated. The experimental results demonstrate that these features not only enable the identification of mask wearing, but also allow for the determination of the specific mask type. Unlike traditional 2D images that are susceptible to the changes in ambient lighting, the developed mask recognition system employs a ToF depth camera to capture the depth images. Consequently, these depth images prove to be robust to variations in lighting conditions and are capable of operating reliably in low-light environments. By extracting local features from the facial depth image, specifically focusing on the contour of the central side shadow line of the face, the system achieves rapid and accurate recognition of mask wearing. This advantage renders it suitable for deployment in various scenarios that require swift identification, such as large-scale performance venues, security checkpoints in public transportation, and entrances and exits of medical facilities.

**Figure 8:**
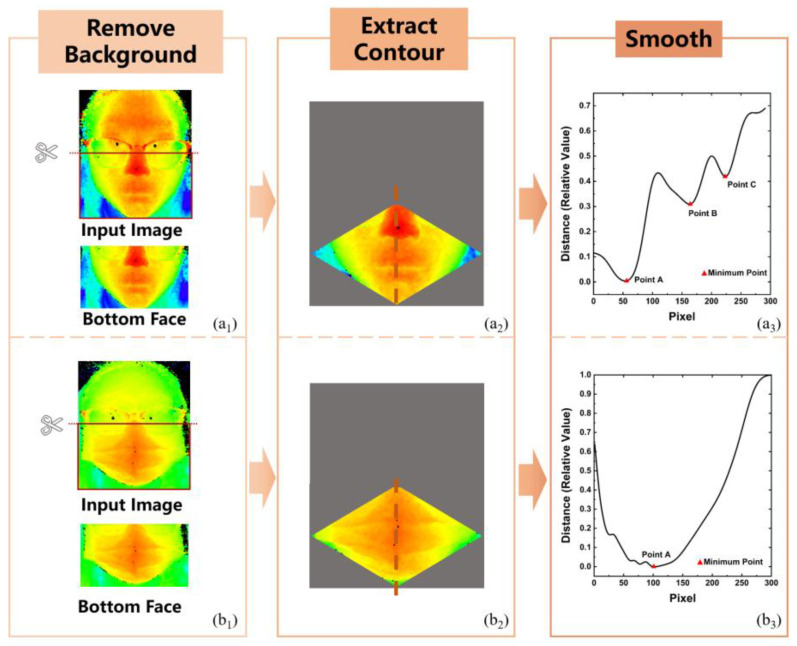
Overview of facial contour extraction. (**a_1_**) Remove background of the image without a mask; (**a_2_**) Extract contour of the image without a mask; (**a_3_**) Smooth contour curve of the image without a mask; (**b_1_**) Remove background of the image with a surgical mask; (**b_2_**) Extract contour of the image with a surgical mask; (**b_3_**) Smooth contour curve of the image with a surgical mask [97].

Figure 8 visually depicts the overview of facial contour extraction. The process begins with the data acquisition module, where a ToF camera captures a depth image of the face. Subsequently, facial contours are extracted from the depth image to serve as recognition features. The classification process primarily relies on feature descriptors, which encompass spatial feature descriptors extracted directly from the facial center silhouette contour (FCSC) and frequency feature descriptors obtained through Fourier transform.

The system runs on a regular laptop (Intel Core AMD Ryzen 7 6800H CPU running at 3.2 GHz, and 16 GB of RAM) and MATLAB is used for image processing. The average processing time of the algorithm is 32 ms, which corresponds to 31.55 FPS. Table 2 shows the comparison of this work with other works. Currently, there is less related work on mask recognition based on depth images and most of the work is based on RGB images. However, depth images captured by low-cost ToF cameras are less affected by ambient light than RGB image-based methods. Therefore, the accuracy of [98] has an advantage over these works. Most of the works with high recognition accuracy based on RGB cameras are based on well-established DL methods. However, this method is more interpretable, less computationally expensive, and computationally faster than most network-based methods.

## 4. Discussion

This review systematically examines mask detection and recognition techniques, including in-depth analyses of datasets (Section 2) and algorithmic approaches (Section 3). Building on the previous sections, key issues and challenges are discussed and summarized, including the technical evolution of the methods, the comparative evaluation of different detection methods, and considerations for multimodal integration and deployment.

### 4.1. Coexistence of “Face-Detection-and-Classification” and “Object-Detection-Models”

In face mask detection tasks, the two paradigms—”feature-extraction-and-classification-based” and “object-detection-models-based”—each exhibit unique applicability and advantages. Early research on face detection was relatively mature, leading some researchers to extend it naturally to face mask detection as a facial attribute recognition problem. This approach involves using specialized face detection algorithms to locate faces and subsequently identify the mask status within the cropped face region. This has proved particularly effective and intuitive for analyzing mask-wearing details (e.g., whether the nose is covered or if the mask is tilted) and integrating facial attributes such as expressions or keypoints. However, when applied to scenarios involving dense crowds or real-time processing requirements, this approach places high demands on the accuracy and speed of the face detection module.

Meanwhile, the rapid advancements in general object detection technologies have given rise to another viable approach—treating “face mask-wearing individuals” as a direct detection target, thereby completing localization and classification in a single step. Compared with the two-stage process of face detection followed by classification, this method is better suited for complex scenarios involving multiple objects and scales. It also benefits from the optimization and updates in mainstream object detection frameworks (e.g., breakthroughs in real-time detection with YOLO and SSD series models, and the high-resolution precision of Faster R-CNN in detailed detection scenarios). Additionally, these methods are highly compatible with attention mechanisms, lightweight networks, or multimodal data (e.g., infrared or depth images), further enhancing the flexibility and accuracy of the detection system.

In summary, “feature-extraction-and-classification-based” and “object-detection-models-based” approaches each possess distinct strengths that cater to different application needs while allowing for potential complementarity. The former is more intuitive for fine-grained mask-wearing analysis and facial attribute integration, while the latter excels in multi-target parallel detection, adaptability to complex scenarios, and alignment with the evolution of general detection technologies. In practical applications, the choice between these paradigms—or their hybridization—depends on specific scenario requirements, computational resources, and the level of detail required for mask-wearing analysis.

In the task of face mask detection, the two primary approaches—”face detection followed by classification” and “one-step object detection”—reflect differences in technical pathways and trade-offs across accuracy, speed, and scalability.

(1) Advantages and limitations of face detection and classification: This two-stage approach excels in scenarios requiring fine-grained analysis of face mask usage. In medical settings, for instance, high protective standards necessitate precise evaluations of whether medical or N95 masks adequately cover the nose and mouth. This method allows for more detailed annotation of facial regions and associated features. However, it relies heavily on the reliability of the face detection module; any errors in face localization can directly affect the subsequent classification accuracy, leading to reduced overall precision or increased false positives. Additionally, in densely populated environments, the computational burden of sequentially detecting and classifying faces frame by frame poses challenges to real-time processing, necessitating optimization in network architecture or inference speed.
(2) Flexibility of one-step object detection methods: Treating masked faces as a category within general object detection enables face mask detection to leverage the latest advancements in object detection. Single-stage detectors, known for their high inference speeds, are well-suited for scenarios requiring real-time monitoring, such as surveillance systems in train stations, airports, and shopping malls. Two-stage detectors, on the other hand, excel in high-precision applications, making them suitable for scenarios demanding detailed analysis. This “one-step” detection approach offers significant advantages in handling multi-target and multi-scale scenarios. Additionally, it integrates seamlessly with emerging technologies such as attention mechanisms and transformer architectures and benefits from pre-training on large-scale general datasets, achieving strong generalization even on smaller face mask datasets.
(3) Balancing speed, accuracy, and hardware resources: Both two-stage and one-stage methods require a careful balance between speed, accuracy, and resource efficiency. In resource-constrained environments, such as embedded devices, lightweight optimization techniques, like model pruning, quantization, and knowledge distillation, can significantly reduce computational overhead. Pruning and quantization compress network structures and represent model parameters with lower bit-widths, improving inference speed. Knowledge distillation enables a teacher model to transfer feature representations to a student model, maintaining high accuracy while reducing model size.
(4) Scalability and multi-task integration: Face mask detection is often combined with other tasks, such as face recognition or behavior analysis. The two-stage approach allows for additional classification or regression modules to be stacked on cropped ROIs, while one-step detection methods can leverage multi-task learning to simultaneously predict masks and other attributes or targets. However, increasing the number of tasks raises model complexity, requiring trade-offs between interpretability, real-time performance, and resource consumption.
(5) Future research directions: Future research may focus on few-shot learning and incremental learning to quickly adapt to new face mask types. Domain adaptation and transfer learning approaches can enhance model generalization across varying environments, such as differing camera setups or lighting conditions. Furthermore, ensuring robust performance while addressing privacy protection and fairness concerns remains critical. Balancing detection efficiency with minimal invasiveness in privacy-sensitive applications, and ensuring equitable representation across diverse demographic groups in datasets, are essential priorities.

In summary, both “face detection followed by classification” and “one-step object detection” have their unique advantages. The choice between these approaches depends on specific application requirements. Through the incorporation of lightweight optimization, multi-task learning, and cross-domain adaptation, future research can further extend the applicability and robustness of face mask detection technologies.

### 4.2. Diversity and Application Requirements of Datasets

The dataset plays a decisive role in shaping the research direction and practical efficacy of algorithms. For methods based on face detection and classification, researchers typically require high-quality face detection data to ensure the accuracy and stability of face mask recognition after cropping the facial regions. Furthermore, if the application scenario emphasizes evaluating whether a face mask is worn correctly, datasets must include detailed annotations regarding mask coverage and wearing styles. Such high-precision annotations are often time-consuming and demand significant expertise from annotators. An insufficient dataset scale may result in model biases toward certain wearing types or demographic characteristics (e.g., race, age), thereby limiting its generalization to broader scenarios.

In contrast, methods based on object detection prioritize diverse annotations across multiple scenes and scales. When face mask detection is incorporated into the general object detection framework, the dataset requirements increase significantly in both volume and diversity in order to enable the effective learning of small- and large-scale facial targets. Scenarios with dense crowds, complex backgrounds, or severe occlusions particularly require rich scene samples to ensure the robustness and generalization of models in real-world environments. For example, in locations such as subway stations or stadiums, where lighting conditions vary and human traffic is dense, models trained without adequate scene-specific samples may exhibit high false-negative or false-positive rates. Additionally, if the goal is to simultaneously evaluate mask-wearing correctness, more granular annotation strategies are necessary. These would subdivide the “masked face” category into “correctly worn” and “incorrectly worn” states, and potentially further differentiate specific errors such as mask slippage, covering only the mouth, or exposing the nose.

The combination of different data sources has also significantly expanded the application potential of face mask detection. Real-world images reflect environmental variability and potential noise factors, such as changes in lighting, cluttered backgrounds, and diverse human postures. Models that perform well on such data tend to exhibit higher credibility when deployed in practical scenarios. Meanwhile, synthetic data can rapidly expand dataset size and provide models with a variety of mask shapes, textures, and color variations. However, synthetic data often suffer from distribution bias or incomplete alignment with real-world scenarios. If improperly combined with real data, this may limit the model’s performance in practical applications. Consequently, researchers often adopt mixed or progressive training strategies, leveraging the diversity and controllability of synthetic data while using a small amount of real data for calibration or fine-tuning to enhance the model’s cross-domain adaptability.

The introduction of multimodal data (e.g., infrared and depth images) further enhances the adaptability of face mask detection in specialized scenarios. In environments with poor lighting or severe interference, relying solely on visible light often fails to yield stable detection results. Infrared imaging or depth sensing can provide additional structural or thermal radiation information, helping models distinguish real faces from fake masks and detect mask edges and coverage. This is particularly critical in high-standard medical protection settings or at security checkpoints. However, multimodal data collection faces challenges such as hardware costs, synchronization calibration, and the complexity of data fusion algorithms. If dataset annotations are incomplete or lack cross-modal alignment, model performance and deployment efficiency may also be adversely affected.

In summary, the selection and construction of datasets must balance three critical factors—scale, annotation quality, and diversity—while aligning with the technical approaches and application objectives of the algorithms. To address the increasingly complex demands of face mask detection, future efforts are likely to focus on the following areas:

(1) Refinement of annotation schemes: Beyond merely distinguishing between “correct” and “incorrect” mask wearing, further distinctions should be made regarding mask types, levels of occlusion, and related attributes. Such detailed annotations would better support high-precision or interpretable applications.
(2) Cross-domain integration and scenario coverage: Collecting more representative image data from diverse domains, such as urban transportation, medical protection, and industrial environments, while leveraging synthetic data for targeted transfer learning and generalization testing, will enhance the adaptability of models across varied application scenarios.
(3) Privacy and fairness considerations: Striking a balance between the need to detect critical facial regions and protecting individual privacy is essential. Additionally, ensuring balanced representation of different races, genders, and age groups within datasets will mitigate systemic biases and prevent unintended disparities in real-world deployments of face mask detection systems.

### 4.3. Multimodal Fusion and Boundary Challenges

Single-modal image detection methods often face significant limitations in complex environments, such as variations in lighting, severe occlusions, and background clutter. These factors can lead to performance degradation in traditional face mask detection models based solely on visible-light images. Multimodal fusion technologies, which integrate data from various sensors (e.g., depth cameras, infrared imaging, thermal imaging), provide additional feature dimensions that significantly enhance the robustness and accuracy of models in challenging scenarios.

Infrared imaging, by capturing thermal radiation features, can compensate for the deficiencies of visible-light imaging in low-light or high-glare conditions. For instance, in nighttime settings, subway stations, or strongly backlit environments, traditional RGB images may fail to capture clear facial features, while infrared images offer supplementary thermal distribution information, facilitating more precise localization of face mask regions. Additionally, thermal maps can reveal abnormal temperature distributions, providing valuable contextual data for epidemic monitoring.

Depth images, generated by sensors such as ToF cameras or structured light systems, offer three-dimensional geometric information that aids in distinguishing real faces from counterfeit coverings, such as printed masks or photographs. This capability is especially critical in high-security scenarios, where preventing fraudulent attacks is paramount. Moreover, depth data can analyze the spatial relationship between the face and the mask, enabling the evaluation of mask-wearing correctness, such as coverage completeness or signs of slippage.

By combining texture information from RGB images, thermal features from infrared data, and geometric insights from depth images, multimodal systems enable comprehensive analysis of face mask-wearing status. Such fusion techniques are particularly effective in handling complex backgrounds, uneven lighting, or densely populated scenarios.

Despite the significant potential of multimodal fusion, its widespread application still faces several key challenges:

(1) Hardware costs and system complexity: Multimodal systems typically require multiple sensors (e.g., RGB cameras, depth cameras, infrared cameras) to work in tandem. The hardware acquisition costs for such systems are substantially higher than those for single-modal systems. Additionally, to ensure temporal and spatial alignment among multiple sensors, high-precision synchronization mechanisms and dedicated calibration algorithms are required. These demands not only increase system complexity but also raise operational and maintenance costs.
(2) Data fusion and computational efficiency: Multimodal data differ significantly in terms of physical properties, resolution, frame rate, and data formats, making the fusion process highly complex. Effective fusion strategies must address cross-modal alignment issues, such as spatial overlapping between depth and RGB images, while maintaining computational efficiency. For instance, directly inputting multimodal data into multi-stream CNNs or transformer-based models may lead to excessive resource requirements, making real-time applications infeasible. To address this, researchers have proposed strategies such as feature-level fusion and decision-level fusion. These methods integrate multimodal information either during feature extraction or at the classification stage. However, the choice of fusion method often requires balancing precision and speed based on the application scenario.
(3) Annotation and data scarcity: Multimodal datasets require annotations across multiple dimensions, and semantic consistency among modalities must be ensured, which increases the cost and complexity of dataset construction. Furthermore, modalities such as infrared and depth imaging are not yet widely used in real-world applications, resulting in a scarcity of publicly available multimodal datasets. This limitation constrains the training and evaluation of multimodal models and may reduce their generalizability in real-world scenarios.

## 5. Conclusions

This review provides a comprehensive analysis of face mask detection and recognition techniques, offering insights into datasets, methodologies, and challenges within the field. The paper systematically categorizes detection approaches into two primary paradigms—“Feature-Extraction-and-Classification-Based Methods” and “Object Detection Models-Based Methods”—and highlights their respective workflows, strengths, and limitations. It also emphasizes the importance of datasets, addressing the critical role of scale, annotation granularity, diversity, and cross-modal integration in enhancing algorithm performance and adaptability.

Additionally, the review examines the advancements in multimodal fusion technologies, including the use of infrared and depth imaging, which significantly improve detection robustness in complex environments characterized by poor lighting, occlusions, and cluttered backgrounds. The challenges associated with multimodal fusion—such as sensor synchronization, data fusion complexity, computational overhead, and dataset scarcity—are also discussed, alongside potential strategies to overcome these hurdles. By delving into the technical evolution of detection methods, the paper underscores the trade-offs between speed, accuracy, and scalability, providing a clear comparison between two-stage and one-stage detection approaches.

In conclusion, this review highlights several promising directions for future research, including the refinement of annotation schemes to support more detailed and nuanced analyses, the incorporation of diverse data sources to improve model generalization, and the development of advanced multimodal frameworks to address the complexities of real-world applications. These efforts are anticipated to contribute to addressing the evolving challenges in face mask detection, enhancing the robustness and reliability of models, and promoting equitable and privacy-conscious system deployments across diverse scenarios. By synthesizing current advancements and identifying areas requiring further exploration, this review aspires to provide a constructive foundation for advancing research and innovation in the field of face mask detection technologies.

## Figures and Tables

**Table 1:** Summary of COVID-19 related datasets reviewed in this paper.

Dataset	Mask Types	Scales	Annotation Classes	Resolution	Year	Data Link
TFCD	Real	250	2 (masked/unmasked)	320 × 240	2021	https://zenodo.org/records/4739682#.YUmyrrhKgWc (accessed on 6 January 2025)
Kaggle-853	Real	853	3 (masked/not masked/wrongly worn)	Multi	2020	https://www.kaggle.com/datasets/andrewmvd/face-mask-detection (accessed on 6 January 2025)
FMCD	Real	3241	2 (masked/unmasked)	224 × 224	2022	https://github.com/Kyrie-leon/Face-Mask-Classification-Dataset?tab=readme-ov-file (accessed on 6 January 2025)
FaceMask	Real	4866	2 (masked/unmasked)	Multi	2022	https://mvrigkas.github.io/FaceMaskDataset/ (accessed on 6 January 2025)
BAFMD	Artificial	6264	2 (masked/unmasked)	Multi	2022	https://github.com/Alpkant/BAFMD (accessed on 6 January 2025)
AIZOO	Real	7971	2 (masked/unmasked)	Multi	2021	https://github.com/AIZOOTech/FaceMaskDetection (accessed on 6 January 2025)
WearMask	Real	9097	3 (masked/not masked/wrongly worn)	Multi	2020	https://facemask-detection.com/ (accessed on 6 January 2025)
PWMFD	Real	9205	3 (masked/unmasked)	Multi	2021	https://github.com/ethancvaa/Properly-Wearing-Masked-Detect-Dataset (accessed on 6 January 2025)
Kaggle-12k	Real	12,000	2 (masked/unmasked)	Multi	2020	https://www.kaggle.com/datasets/ashishjangra27/face-mask-12k-images-dataset (accessed on 6 January 2025)
Kaggle-FMLD	Artificial	20,000	2 (masked/unmasked)	1024 × 1024	2020	https://www.kaggle.com/datasets/prasoonkottarathil/face-mask-lite-dataset (accessed on 6 January 2025)
MAFA	Real	30,811	Multiple (face frames, mask types)	Multi	2017	https://www.kaggle.com/datasets/revanthrex/mafadataset (accessed on 6 January 2025)
FMLD	Real	41,934	3 (masked/not masked/wrongly worn)	Multi	2021	https://github.com/borutb-fri/FMLD (accessed on 6 January 2025)
RMFRD	Real	92,671	2 (masked/unmasked)	Multi	2020	https://github.com/X-zhangyang/Real-World-Masked-Face-Dataset (accessed on 6 January 2025)
MaskedFace-Net	Artificial	137,016	3 (masked/not masked/wrongly worn)	1024 × 1024	2020	https://github.com/cabani/MaskedFace-Net (accessed on 6 January 2025)
SMFRD	Artificial	500,000	2 (masked/unmasked)	Multi	2020	https://github.com/X-zhangyang/Real-World-Masked-Face-Dataset (accessed on 6 January 2025)

**Table 2:** Comparison of methods for face mask recognition and detection.

Work	Method	Data	Distinguished Type	Accuracy	Efficiency
Cao et al. [98]	YOLOv4-large	2D RGB	With/without Nighttime	94% 77.9%	18 FPS
Nagrath et al. [63]	SSDMNV2	2D RGB	With/without	92.64%	15.71 FPS
Yu et al. [64]	YOLO-v4	2D RGB	With/without	98.3%	54.57 FPS
Walia et al. [15]	ResNet-50	2D RGB	With/without	98%	32 FPS
Jiang et al. [91]	SE-YOLOv3	2D RGB	With/without /Correct wearing	73.7%	15.63 FPS
Su et al. [63]	Transfer learning and efficient-Yolov3	2D RGB	with/without Mask type	96.03%	15 FPS
				97.84%	
Wang et al. [97]	Feature-based	3D Depth	With/without Mask Type	96.9% 87.85%	31.55 FPS

## Data Availability

Data underlying the results presented in this paper are not publicly available at this time but may be obtained from the authors upon reasonable request.
